# Beneficial Role of Blood Flow Restriction Exercise in Heart Disease and Heart Failure Using the Muscle Hypothesis of Chronic Heart Failure and a Growing Literature

**DOI:** 10.3389/fphys.2022.924557

**Published:** 2022-07-06

**Authors:** Lawrence P. Cahalin, Magno F. Formiga, Johnny Owens, Brady Anderson, Luke Hughes

**Affiliations:** ^1^ Department of Physical Therapy, Miller School of Medicine, University of Miami, Miami, FL, United States; ^2^ Departamento de Fisioterapia, Faculdade de Medicina, Universidade Federal Do Ceará, Fortaleza, Brazil; ^3^ Owens Recovery Science, San Antonio, TX, United States; ^4^ Department of Sport, Exercise and Rehabilitation, Northumbria University, Northumbria, United Kingdom

**Keywords:** blood flow restriction, heart disease, heart failure, skeletal muscle, blood flow restricted exercise

## Abstract

**Background:** Blood flow restriction exercise (BFRE) has become a common method to increase skeletal muscle strength and hypertrophy for individuals with a variety of conditions. A substantial literature of BFRE in older adults exists in which significant gains in strength and functional performance have been observed without report of adverse events. Research examining the effects of BFRE in heart disease (HD) and heart failure (HF) appears to be increasing for which reason the Muscle Hypothesis of Chronic Heart Failure (MHCHF) will be used to fully elucidate the effects BFRE may have in patients with HD and HF highlighted in the MHCHF.

**Methods:** A comprehensive literature review was performed in PubMed and the Cochrane library through February 2022. Inclusion criteria were: 1) the study was original research conducted in human subjects older than 18 years of age and diagnosed with either HD or HF, 2) study participants performed BFRE, and 3) post-intervention outcome measures of cardiovascular function, physical performance, skeletal muscle function and structure, and/or systemic biomarkers were provided. Exclusion criteria included review articles and articles on viewpoints and opinions of BFRE, book chapters, theses, dissertations, and case study articles.

**Results:** Seven BFRE studies in HD and two BFRE studies in HF were found of which four of the HD and the two HF studies examined a variety of measures reflected within the MHCHF over a period of 8–24 weeks. No adverse events were reported in any of the studies and significant improvements in skeletal muscle strength, endurance, and work as well as cardiorespiratory performance, mitochondrial function, exercise tolerance, functional performance, immune humoral function, and possibly cardiac performance were observed in one or more of the reviewed studies.

**Conclusion:** In view of the above systematic review, BFRE has been performed safely with no report of adverse event in patients with a variety of different types of HD and in patients with HF. The components of the MHCHF that can be potentially improved with BFRE include left ventricular dysfunction, inflammatory markers, inactivity, a catabolic state, skeletal and possibly respiratory muscle myopathy, dyspnea and fatigue, ANS activity, and peripheral blood flow. Furthermore, investigation of feasibility, acceptability, adherence, adverse effects, and symptoms during and after BFRE is needed since very few studies have examined these important issues comprehensively in patients with HD and HF.

## Introduction

The “muscle hypothesis of chronic heart failure” (MHCHF) attributes the decreased exercise tolerance common in heart failure (HF) to skeletal muscle atrophy and metabolic inefficiency which stimulates a viscous cycle of dyspnea and fatigue; increased ventilation; sympathetic nervous system (SNS) excitation; increased afterload and reduced peripheral blood flow; and a catabolic state all of which worsen cardiac and skeletal muscle performance. (Coats et al., 1994; Coats, 1996; Piepoli and Coats, 2013). Many of the same factors likely contribute to exercise intolerance in heart disease (HD) despite the MHCHF being developed for HF. Many forms of HD elicit many of the factors described in the MHCHF falling within any stage of the HD continuum, but are often less extreme since HF is the end-stage of HD. In fact, HF is defined as a clinical syndrome with the most common characteristics being dyspnea, fatigue, abnormal ventricular filling, and elevated filling pressures resulting in the inability of the heart to pump blood to the body at a rate commensurate with its needs (Coats et al., 1994; Coats, 1996; Piepoli and Coats, 2013).

Blood flow restriction exercise (BFRE) has become a common method to increase skeletal muscle strength and hypertrophy for patients with a variety of orthopedic and other musculoskeletal disorders in whom limited activity, exercise, and workloads are common. (Hughes et al., 2017; Van Cant et al., 2020; Nitzsche et al., 2021). A substantial literature of BFRE in older adults exists in which significant gains in strength and functional performance have been observed without report of adverse events. (Beckwée et al., 2019; Centner et al., 2019; Rodrigo-Mallorca et al., 2021; Labata-Lezaun et al., 2022). Sophisticated tourniquets exist which allow for a more precise and personalized reduction in blood flow based on the limb occlusion pressure enabling safer and more precise BFRE in older adults and in patients with HD and HF. (Weatherholt et al., 2019; Masri et al., 2020; Bordessa et al., 2021; Murray et al., 2021).

The reduction in blood flow and subsequent hypoxia within exercising skeletal muscle during BFRE stimulates anaerobic metabolism and metabolite accumulation promoting rapid muscular fatigue, up-regulated muscle protein synthesis, systemic anabolic hormone release, and possibly angiogenesis. (Loenneke et al., 2012; Pignanelli et al., 2021; May et al., 2022; Reina-Ruiz Á et al., 2022). However, BFR during exercise does elicit a greater increase in hemodynamic response which appears to be less during aerobic exercise compared to resistance training. (Pinto and Polito, 2016; Crisafulli et al., 2018; Pinto et al., 2018; Wong et al., 2018; Silva et al., 2019; Wong et al., 2021). Furthermore, the size of the muscle group appears to influence the hemodynamic response during BFRE with greater muscle groups eliciting a greater hemodynamic response. (Pinto and Polito, 2016; Crisafulli et al., 2018; Pinto et al., 2018; Wong et al., 2018; Silva et al., 2019; Wong et al., 2021). Factors such as mode of exercise, size of muscle group, and BFRE protocol in patients with HD and HF warrants further investigation in view of a limited, but growing literature.

One such BFRE protocol is cuff release after one or more sets of BFRE since there is potential to elicit favorable effects on skeletal muscle and the vasculature including an increase in nitric oxide and endothelium dependent vasodilation due to vascular sheer stress. (Pinto and Polito, 2016; May et al., 2017; Crisafulli et al., 2018; Pinto et al., 2018; Wong et al., 2018; Silva et al., 2019; Wong et al., 2021). In fact, cyclic occlusion and reperfusion via BFRE may promote favorable acute and chronic effects on cardiac and cardiovascular performance in hypertensive subjects and patients with HD and HF. (Shweiki et al., 1992; Higashi and Yoshizumi, 2004; Takano et al., 2005; Horiuchi and Okita, 2012; Pinto and Polito, 2016; May et al., 2017; Crisafulli et al., 2018; Pinto et al., 2018; Wong et al., 2018; Christiansen et al., 2019; Silva et al., 2019; Christiansen et al., 2020; Kambič, 2020; Liu et al., 2021; Wong et al., 2021; Li et al., 2022). Thus, personalized BFRE has the potential to improve skeletal muscle and cardiovascular performance of patients with HD and HF which may subsequently improve many of the MHCHF components and attenuate its viscous cycle. Recent position papers on cardiac rehabilitation suggest that low-load BFRE could be an adjunct exercise in cardiac rehabilitation for patients with HD who are frail with sarcopenia or other musculoskeletal disorders that may prevent moderate to high-intensity resistance training. (Ambrosetti et al., 2020; Hansen et al., 2022). In fact, a recent paper entitled “Is blood flow restriction resistance training the missing piece in cardiac rehabilitation of frail patients?” suggested that BFRE be started in the early phases of cardiac rehabilitation followed by moderate to high-intensity resistance training. (Kambic et al., 2022).

The purpose of this paper is to provide a comprehensive overview of the MHCHF and how BFRE may affect each component of the original and revised MHCHF and attenuate its viscous cycle. A systematic review of a growing literature of seven BFRE studies in HD and two BFRE studies in HF will follow in which the safety and beneficial effects of BFRE on the pathophysiological manifestations of HD and HF will be highlighted. (Nakajima et al., 2010; Fukuda et al., 2013; Madarame et al., 2013; Tanaka and Takarada, 2018; Groennebaek et al., 2019; Ishizaka et al., 2019; Kambič et al., 2019; Kambič et al., 2021; Ogawa et al., 2021). Recent results from studies we have performed showing the beneficial effects of BFRE on cardiac and cardiovascular performance in patients with HF will also be presented. (Mostoufi et al., 2020; Gempel et al., 2022; Johnson et al., 2022). The paper will conclude with suggestions to safely perform BFRE in HD and HF using the currently available literature of BFRE in HD and HF.

## The Muscle Hypothesis of Chronic Heart Failure and Blood Flow Restriction Training

Patients with HF suffer from marked dyspnea and fatigue because of the inability of the heart to pump blood adequately to peripheral tissues resulting in excessive blood volume in the chambers of the heart and insufficient blood flow to skeletal muscles. (Coats et al., 1994; Coats, 1996; Piepoli and Coats, 2013). Insufficient blood flow to the skeletal muscles results in limited exercise tolerance, marked dyspnea and fatigue, further skeletal muscle weakness, and possibly a skeletal muscle metabolic myopathy. (Coats et al., 1994; Coats, 1996; Piepoli and Coats, 2013). Additionally, the excessive blood volume in the chambers of the heart results in a poorer capacity of the heart to pump blood to the periphery. (Christiansen et al., 2019; Li et al., 2022). In fact, the excessive blood volume in the cardiac chambers is a major target of pharmacologic treatment for HF and includes the administration of diuretics and vasodilators to decrease the excessive blood volume by reducing venous return and increasing peripheral vasodilation. (Coats et al., 1994; Coats, 1996; Piepoli and Coats, 2013).

**FIGURE 1:**
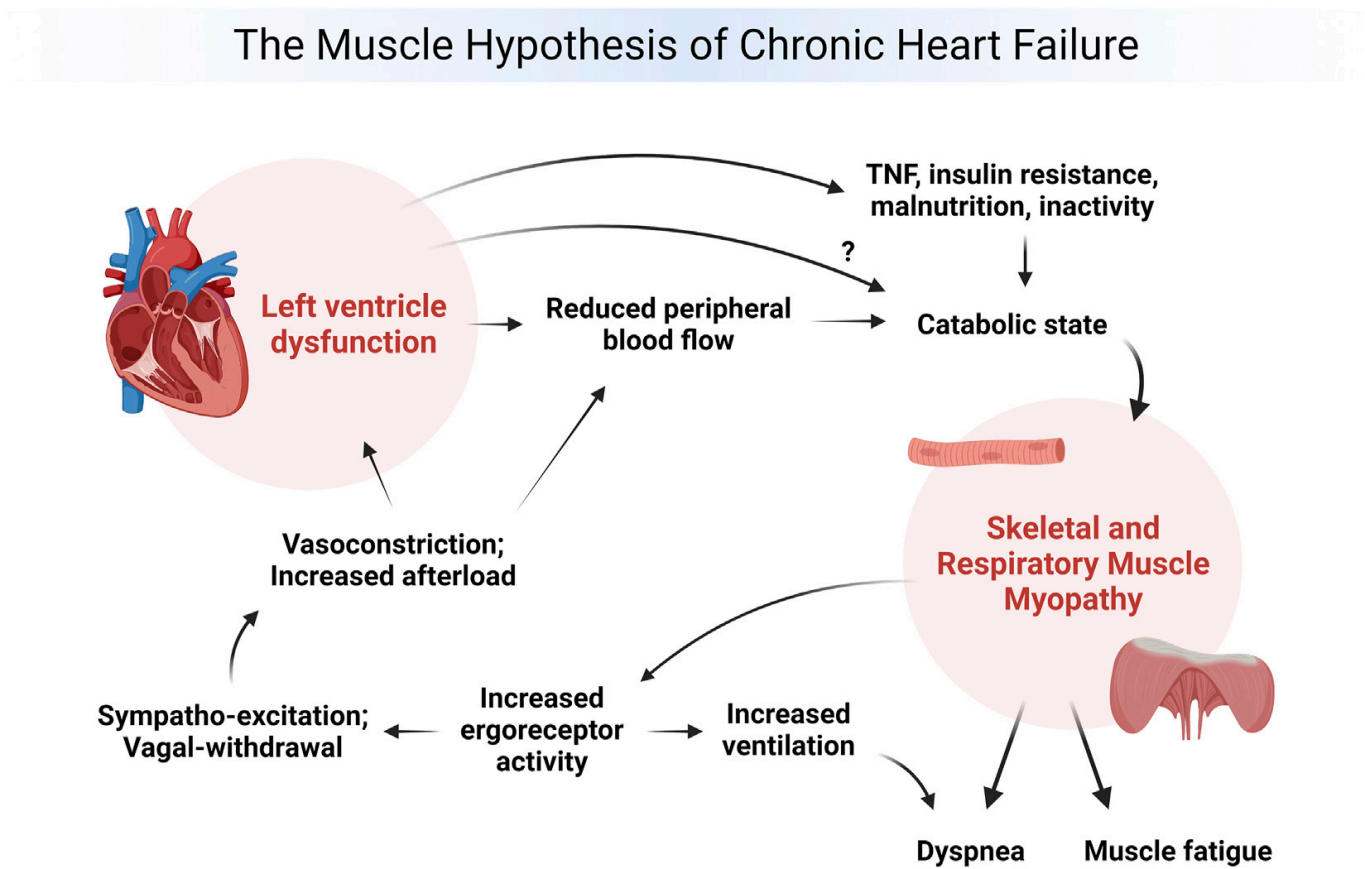
The muscle hypothesis of chronic heart failure (created with BioRender).

The manner by which left ventricular dysfunction contributes to skeletal muscle weakness and many pathophysiological manifestations of heart failure have been keenly described in the MHCHF. This conceptual model outlines the major ramifications from HF on the body and identifies the major role that skeletal muscle weakness plays in worsening HF (Figure 1). As shown in Figure 1, left ventricular (LV) dysfunction due to HF initiates a viscous cycle of detrimental effects on the body including a reduction in peripheral blood flow, inactivity and elevated inflammatory markers, a catabolic and skeletal muscle myopathy including the respiratory muscles (contributing to the marked dyspnea and fatigue described above), and increased ventilation, SNS activity, and peripheral vasoconstriction all of which further worsen LV function and HF. (Coats et al., 1994; Coats, 1996; Piepoli and Coats, 2013). An improvement in skeletal muscle strength in HF may improve many of the pathophysiological manifestations of HF outlined above. (Coats et al., 1994; Coats, 1996; Piepoli and Coats, 2013).

In view of the potential effects of BFRE on the vasculature and cardiovascular function, it is possible that BFRE may be a potential method to improve not only skeletal muscle strength and endurance and exercise tolerance, but possibly even the pumping ability of the heart in HF. (Shweiki et al., 1992; Coats et al., 1994; Coats, 1996; Halliwill, 2001; Higashi and Yoshizumi, 2004; Takano et al., 2005; Chen and Bonham, 2010; Rossow et al., 2011; Horiuchi and Okita, 2012; Piepoli and Coats, 2013).

The three key mechanisms by which BFRE could potentially improve each area within the MHCHF include increasing skeletal muscle strength, decreasing venous return, and improving peripheral vasodilation (Figure 1). Since patients with HF suffer from marked dyspnea and fatigue, BFRE may provide an alternate form of exercise producing less dyspnea and fatigue while promoting greater muscle strength in a shorter period of time with a less frequent and intense exercise prescription. Additionally, the work of breathing during BFRE appears to increase which may facilitate a mild to moderate form of respiratory muscle training and attenuate the respiratory muscle metaboreflex. (May et al., 2017; Crisafulli et al., 2018). Decreasing venous return has the potential to improve cardiac filling pressures and LV dysfunction which alone could attenuate many of the pathophysiological manifestations of HF (Figure 1) while increasing skeletal muscle strength. The hypoxic state created during BFRE appears to up regulate hypoxia-inducible factor 1alpha (HIF-1A) which in turn promotes gene expression of vascular endothelial growth factor (VEGF) and promote angiogenesis. (Shimizu et al., 2016). Increased angiogenesis in the extremities may improve fluid flow dynamics and hypertension in patients with HF. In elderly individuals, 4-weeks of low intensity leg press with BFR significantly increased lower leg capillarity. (Patterson and Ferguson, 2011). Lastly, increasing endothelium-dependent vasodilation through BFR exercise is the same mechanism physicians use when treating HF by using a variety of pharmacologic agents. Therefore, BFRE appears to be a potential therapeutic modality to counter the viscous cycle of HF for which reason the below systematic review was performed.

Also, although the MHCHF was specifically designed for patients with HF, many of the components outlined in the original and revised MHCHF may still be present in patients with HD and other disorders such as chronic obstructive pulmonary disease and cachexia. (Coats et al., 1994; Coats, 1996; Piepoli and Coats, 2013). Examples of pathophysiologic manifestations in such patients include a reduction in cardiac output, inflammation, systemic catabolism, immobilization and deconditioning, autonomic nervous system abnormalities as well as skeletal muscle structural, metabolic, and functional abnormalities. Of course, the abnormalities in HF are much more profound, but as in HF, patients with HD may also have significant improvements in one or more of the above components of the MHCHF with BFRE.

## Methods

A comprehensive literature review was performed in PubMed and the Cochrane library through February 2022. Supplementary Appendix S1 presents the complete search strategy which was conducted in English and included a mix of terms for the key concepts *blood flow restriction*, *heart disease*, *heart failure*, *physical function* and *skeletal muscle*. The reference list of eligible studies was also screened to identify other potentially relevant publications.

A study had to meet the following criteria to be included in the systematic review: 1) the study was original research conducted in human subjects older than 18 years of age and diagnosed with either heart disease or heart failure, 2) study participants performed BFRE, and 3) post-intervention outcome measures of cardiovascular function, physical performance, skeletal muscle function and structure, and/or systemic biomarkers were provided. Exclusion criteria included review articles and articles on viewpoints and opinions of BFRE, book chapters, theses, dissertations, and case study articles. Studies were only considered for eligibility if they have been peer reviewed and published prior to the search. Study quality was assessed using two separate instruments including the TESTEX scale for randomized controlled trials (RCTs) (Smart et al., 2015) and the National Institute of Health quality assessment tool for before-after (Pre-Post) studies with no control group. (NIH, 2021).

This study was conducted according to the Preferred Reporting Items for Systematic Reviews and Meta-Analyses (PRISMA) guidelines.

## Results

**FIGURE 2:**
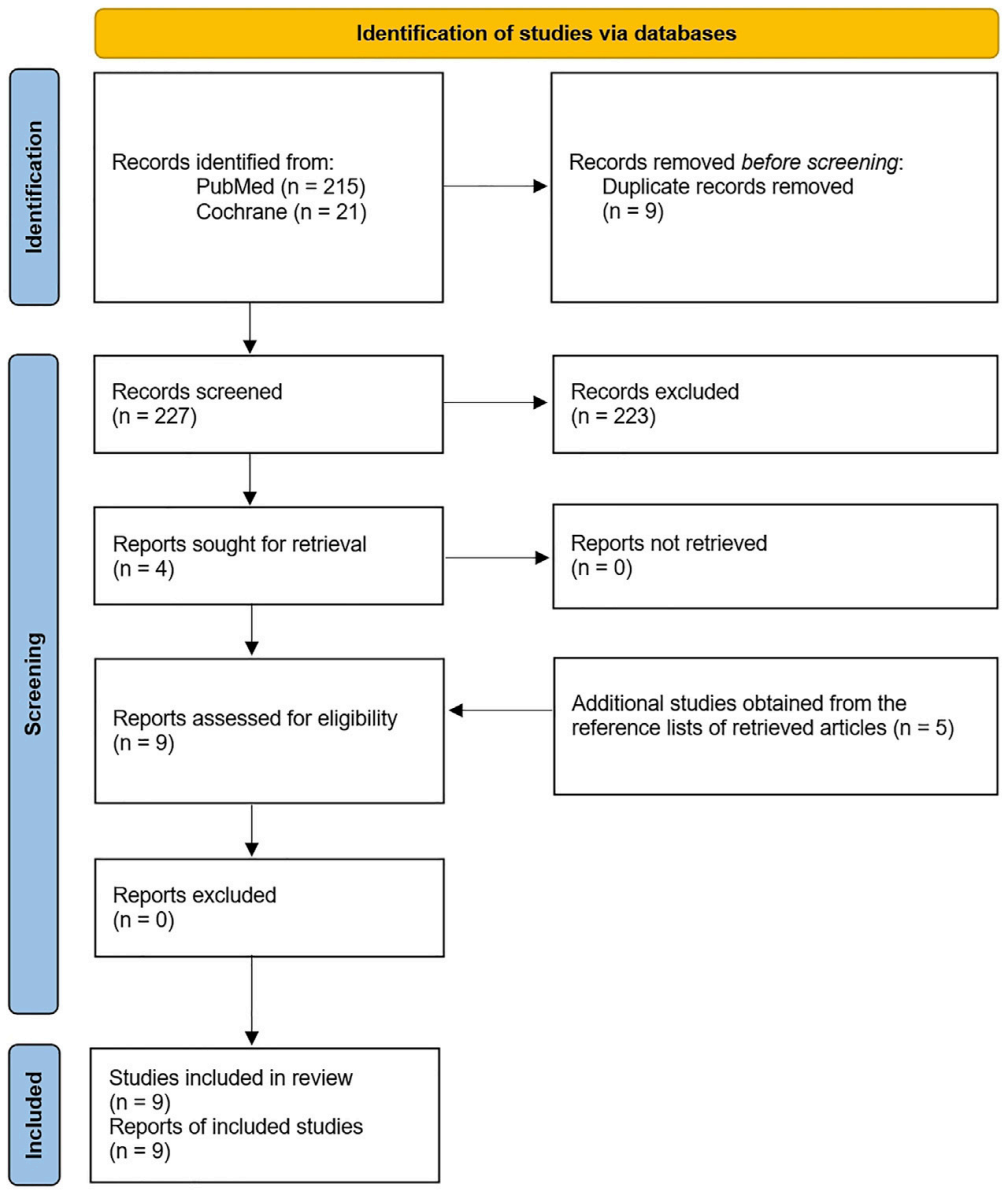
Flow diagram of study selection.

The search identified a total of 227 papers of which 9 met our inclusion criteria (7 reports of BFRE in HD and 2 reports of BFRE in HF) (Figure 2). The assessment of study quality revealed that 3 of the 5 RCTs were of a high quality with high reporting criterion while the other two RCTs were of modest quality and reporting criterion (Table 1). The assessment of study quality of the pre-post studies without a control group revealed that 3 of the studies were of a fair quality and one was of a good quality, but almost all studies were observed to have one or more areas of assessment that were either unable to determined, not reported, or not applicable (Table 2). A systematic review of each study will be provided below beginning with the studies that have examined the effects of BFRE in HF.

**TABLE 1:** TESTEX assessment of the quality and reporting of included randomized controlled trials.

	Study Quality Criterion						Study Reporting Criterion											
Study	1	2	3	4	5	Total	6a	6b	6c	7	8a	8b	9	10	11	12	Total	Overall Total
Tanaka and Takarada (2018)	1	1	0	1	0	3	0	1	0	0	1	1	1	0	0	1	5	8
Groennebaek et al. (2019)	1	1	0	1	1	4	1	1	1	1	1	1	1	0	1	1	9	13
Kambic et al. (2019) & Kambic (2020)	1	1	1	1	0	4	1	1	1	1	1	1	1	0	1	1	9	13
Ogawa et al. (2021)	1	0	0	1	0	2	0	1	0	0	1	1	1	0	1	1	6	8

**TABLE 2:** National Institute of Health quality assessment of before-after (Pre-Post) studies with no control group of included studies.

	Itens													
Study	1	2	3	4	5	6	7	8	9	10	11	12	Total	QR
Nakajima et al. (2010)	1	0	1	CD	0	1	1	NR	NA	1	0	1	6	Fair
Madarame et al. (2013)	1	0	1	CD	0	1	1	NR	NA	1	0	1	6	Fair
Fukuda et al. (2013)	1	0	1	CD	0	1	1	NR	NA	1	1	1	7	Fair
Ishizaka et al. (2019)	1	1	1	1	0	1	1	NR	NA	1	1	1	9	Good

CD, cannot determine; NR, not reported; NA, not applicable; QR, quality rating.

### Blood Flow Resistance Training in Heart Failure

Both of the studies of BFRE in HF were RCTs and were performed without report of adverse events and observed significant improvements in several of the MHCHF components (Table 3). (Tanaka and Takarada, 2018; Groennebaek et al., 2019) The first study of BFRE in HF examined the effects of 6 months of bilateral aerobic BFRE with cycling performed at 40–70% of peak oxygen consumption for 15 min, 3x/week in 30 patients with both reduced and preserved ejection fraction heart failure who were randomized to either BFRE or control group. (Tanaka and Takarada, 2018). Pneumatic cuffs were placed proximally on both thighs and inflated to a pressure 40–80 mmHg above systolic blood pressure (mean ± SD of 208.7 ± 7.4 mmHg) and remained inflated during the entire cycling session. The control group performed the same intensity and duration of aerobic cycling, but without BFR. After the 6-months study period both the BFRE and control group increased skeletal muscle strength and endurance reflected by increased Watts during cycle ergometry, but the BFRE group had a significantly greater increase in oxygen consumption compared to the control group (approximately 40 versus 10%, respectively). Other changes in the BFRE group that were not observed in the control group included a significant decrease in brain-natriuretic peptide (BNP) and C-reactive protein as well as a significant increase in oxygen consumption at the anaerobic threshold. Importantly, the improvement in BNP was significantly correlated in a negative direction to the improvement in peak oxygen consumption meaning that a greater reduction in BNP was associated with greater levels of peak oxygen consumption. (Tanaka and Takarada, 2018). The improvement in BNP and significant negative relationship with improvement in peak oxygen consumption is suggestive of improved cardiac performance due possibly to decreased preload from BFRE. (Takano et al., 2005; Tanaka and Takarada, 2018). Similar changes in BNP were also observed by Passino et al. after 9 months of aerobic exercise performed at 65% of the peak oxygen consumption heart rate 3x/week in patients with reduced ejection fraction heart failure, but without BFRE.^59^ Also observed by Passino were significant favorable decreases in end-systolic and diastolic volume after the 9-months program as well as an identical significant relationship between BNP and peak oxygen consumption. (Passino et al., 2006). Although cardiac performance in the first study of BFRE was not examined, the above results of Passino et al. suggest that similar improvements in cardiac performance may have occurred in the Tanaka and Takarada study. (Passino et al., 2006).

**TABLE 3:** Studies of blood flow restriction training in patients with heart failure.^a^

Author	Sample	Outcome measures	Procedures	Results
Tanaka and Takarada (2018)	30 male patients with both reduced and preserved EF heart failure due to MI with baseline EF, BNP, BUN, Creatinine, and eGFR in the BFR and non-BFR groups of 49.3 *vs*. 54.4, 148.1 *vs*. 144.5, 16.6 *vs*. 17.6, 1.0 *vs*. 1.0, 62.0 *vs*. 65.0, respectively. Patients were randomly assigned to the BFR group or control group performing aerobic exercise, but without BFR. Medications included ACE-I/ARB, Beta-blockers, Aldosterone antagonists, and statins with equal administration between groups except in ACE-I/ARB with a greater number of patients in the control group receiving ACE-I	Peak VO2, VO2 @AT, BNP, CRP, thigh circumference	Chronic (3x/week for 24 weeks) assessment of Aerobic BFR Ex. performed at 40–70% of peak VO2/W for 15 min/session. Aerobic BFR exercise was performed with pneumatic cuffs (90 mm wide and 700 mm in length) placed on the proximal ends of the thighs and inflated to a mean pressure of 208.7 mm Hg	No adverse events were reported. Peak VO2, VO2@AT, BNP, and CRP were significantly improved in the BFR Ex. group
Groennebaek et al. (2019)	36 male patients with heart failure reduced EF were randomly allocated to BFR, RIPC, or a control group receiving no intervention with respective EF of 35, 37, and 35%. Baseline BNP and eGFR in the BFR, RIPC, and control groups were 518, 297, and 188, respectively and 79, 84, and 89, respectively. Medications included ACE-I/ARB, Beta-blockers, sacubitril/valsartan, mineralocorticoids, diuretics, platelet inhibitors, and statins with equal administration between groups	Isometric strength, 6 MWT distance ambulated, QOL, skeletal muscle mitochondrial function	Chronic (3x/week for 6 weeks) assessment of Resistance BFR Ex. performed at 30% 1 RM with 50% of LOP while performing 4 sets of bilateral knee extension exercises with pneumatic cuffs inflated throughout the training period. RIPC was administer 3x/week for 6 weeks and consisted of 4 cycles of 5 min of upper arm ischemia followed by 5 min of reperfusion	No adverse events were reported. BFR Ex. significantly improved maximum isometric strength, 6 MWT distance ambulated, QOL, and mitochondrial function

a The design of both studies of BFRE, in HF, were RCTs.

The above studies highlighting a possible improvement in cardiac performance from exercise with and without BFRE prompted us to examine via echocardiography the acute effects of BFRE in two patients with HF one of whom had severe HF (LVEF = 25%) and the other with less severe HF (LVEF = 65%). (Johnson et al., 2022). A series of echocardiograms were obtained at rest and during 15 alternating straight leg raises (SLR) of each lower extremity performed supine without added resistance, with and without BFRE at a limb occlusion pressure of 60%. The key outcome measures included LVEF, stroke volume, and cardiac index with the hypothesis that improvements in the above measures would be observed in the patient with severe HF, but not in the patient with less severe HF. The results of the study found all outcome measures decreased in the patient with less severe HF, but improvements in all outcomes were observed in the patient with severe HF with an improvement in the cardiac index of almost 70%. (Johnson et al., 2022). The improvement in cardiac index of almost 70% was observed during SLR with BFRE and suggests an improvement in cardiac performance as well as peripheral blood flow.

In view of the above favorable changes in cardiac performance from BFRE in the patient with severe HF, chronic BFRE was performed via SLR without added resistance 2x/week for 3 weeks at 60% limb occlusion pressure. (Gempel et al., 2022). Three sets of 15 alternating SLR of each leg were performed in supine and followed by deflation of the cuffs after each set for a period of 5 min. The patient suffered from gastrointestinal distress before BFRE while participating in cardiac rehabilitation which appeared to be worsened by BFRE for which reason BFRE was terminated after 3 weeks. Despite this, the patient was observed to have a 50% improvement in SLR ability as well as a 13 and 12.5% increase in knee extensor and hip flexor strength, respectively, without change in cardiac performance. (Gempel et al., 2022). Additionally, the average increase in heart rate, systolic and diastolic blood pressure was 15 bpm, 10 mmHg, and 2 mmHg, respectively, with average Borg RPE, modified dyspnea, and lower extremity pain scores of 12/20, 1/10, and 7/10, respectively. No adverse events were observed during the above two echocardiographic studies. (Gempel et al., 2022; Johnson et al., 2022).

The second study of BFRE in HF was performed by Groennebaek et al. in which 36 patients with reduced ejection fraction heart failure (LVEF = 35–37%) were randomized to BFRE, remote ischemic preconditioning (RIPC), or non-treatment control (Table 3). (Groennebaek et al., 2019) BFRE and RIPC were performed 3x/week for 6 weeks. BFRE consisted of resistance exercise performed at 30% of 1 RM with 50% limb occlusion pressure during which 4 sets of bilateral knee extension exercise separated by 30-s rest periods with a pneumatic cuff inflated until the 4 sets were completed. The RIPC protocol consisted of 4 cycles of 5 min upper arm ischemia followed by 5 min of reperfusion. The results of the study found BFRE produced significant improvements in maximal isometric strength, mitochondrial function, 6-min walk test (6 MWT) distance ambulated, and quality of life which were not observed in the RIPC or control groups. Thus, in view of the above results, patients with HF performing aerobic exercise, functional exercise, and resistance training improve skeletal muscle strength and endurance, mitochondrial function, oxygen uptake, 6 MWT, and quality of life as well as the possibility of improved cardiac function. Furthermore, none of the above studies observed adverse events.

### Blood Flow Resistance Training in Heart Disease

Three of the 7 studies of BFRE in HD were RCTs (Kambic et al., 2019; Kambic, 2020; Ogawa et al., 2021) and the other 4 studies were pre-post studies without control groups. All of the 7 studies examined the effects of resistance BFRE and all were performed without report of adverse events and also observed significant improvements in several of the MHCHF components (Table 4). Five of the 7 studies of BFRE in HD were performed in Japan with all of the 5 studies using KAATSU cuffs bilaterally and with all but one study placing the cuffs on the most proximal portion of the thigh. The other Japanese study placed the cuffs on the most proximal portion of the arms bilaterally and had patients perform 4 sets of bilateral elbow flexion (starting with 30 repetitions followed by 3 sets of 15 repetitions) with and without BFR at 20% of 1 R M and with 30 s of rest between sets. (Fukuda et al., 2013). The 4 other Japanese BFRE studies in patients with HD performed bilateral knee extension at 20% of I-RM starting with 30 repetitions followed by 3 sets of 15 repetitions with and without BFR with 30 s of rest between sets (Madarame et al., 2013) while Ishizaka used the same muscle groups and protocol except that they also examined EMG activity using 10% of 1-RM and provided 5 min of rest between each of the 4 study conditions. (Ishizaka et al., 2019). One of the Japanese studies performed bilateral knee extension and flexion as well as bilateral leg press at 20–30% of 1-RM, 2x/week for 3 months using 30 repetitions followed by 3 sets of 15 repetitions with BFR and with 60 s of rest between sets. (Nakajima et al., 2010). The last Japanese study examined the effects of cardiac rehabilitation with and without BFRE during which BFRE started 5–7 days after surgery if patients were able to walk 200 m and consisted of bilateral knee extension and flexion as well as leg press at 20–30% of 1-RM using 3 sets of 30 repetitions with a 30 s rest between sets. (Ogawa et al., 2021).

**TABLE 4:** Studies of blood flow restriction training in patients with heart disease.

Author	Sample/Study design	Outcome measures	Procedures	Results
Nakajima et al. (2010)	7 stable male patients (mean ± SD age of 52 ± 4 yrs) with IHD (2 post-CABG surgery and 5 post-PTCA). Complete medication use was not reported, but all patients were administered Acetylsalicylic Acid or Ticlopidine Hydrochloride. The study design was a pre-post study without control group	Peak VO2, VO2 @AT, IGF-1, CRP, muscle CSA	Chronic assessment of BFR Ex using 4 sets (30 reps in the 1st set followed by 15 reps in subsequent sets with 60 s of rest between sets) of bilateral leg press, knee extension, and knee flexion at 20–30% of 1 RM 2x/week for 3 months with bilateral BFR (via KAATSU belt at proximal thighs using 100 mmHg cuff pressure initially which was gradually increased to 160–250 mmHg within 2–3 weeks to elicit a Borg RPE score of 16/20)	No adverse events were reported. BFR Ex produced a significantly greater CSA in the quadriceps, hamstring, and adductor muscles with significant increases in leg press, knee extension, and knee flexion 1 RM (approx. 15%) as well as significant increases in peak Watts, Watts @AT, peak VO2, and VO2 @AT. SBP and DBP were unchanged
Madarame et al. (2013)	9 stable patients (7 men, 2 women) with IHD (2 post-CABG surgery and 7 post-PTCA) with a mean ± SD age of 57 ± 6 yrs. Complete medication use was not reported, but patients were not administered anticoagulant drugs. The study design was a pre-post study without control group	HR, noradrenaline, D-dimer, fibrinogen/fibrin degradation products, CRP	Acute and chronic (1-h post Ex) assessment of BFR Ex using 4 sets (30 reps in the 1st set followed by 15 reps in subsequent sets with 30 s of rest between sets) of bilateral knee extension with and without BFR at 20% of 1 RM (via KAATSU belt at proximal thighs using 200 mmHg cuff pressure that was maintained throughout Ex and rest periods)	No adverse events were reported. BFR Ex produced a significantly greater HR and noradrenaline compared to non-BFR Ex. A significantly greater D-dimer and CRP was observed after BFR Ex compared to non-BFR Ex which were no longer statistically significant after plasma volume correction (suggesting that hemoconcentration was responsible for the significant increases in these measures). Plasma fibrinogen/fibrin degradation products were unchanged after both forms of Ex
Fukuda et al. (2013)	6 male patients (mean ± SD age of 69 ± 12 yrs) with IHD (5 post-MI and 1 dilated cardiomyopathy). Medication use was not reported. The study design was a pre-post study without control group	EMG and Borg RPE	Acute assessment of BFR Ex using Thera-Band (medium and light resistance bands) for 4 sets (30 reps in the 1st set followed by 15 reps in subsequent sets with 30 s of rest between sets) of bilateral elbow flexion with and without BFR at 20% of 1 RM (via KAATSU belt at proximal portion of both arms using 110–160 mmHg cuff pressure that was maintained throughout Ex and rest periods)	No adverse events were reported. BFR Ex produced significantly greater EMG and Borg RPE during all sets compared to non-BFR Ex
Kambic et al. (2019) &Kambic (2020)	24 mostly male patients (12/group) with IHD (13 NSTEMI, 11 STEMI) receiving PCI (N = 19) or CABG (*N* = 5) were randomly assigned to a BFR or control group with the control group performing aerobic exercise without BFR. Medication use included ASA and Statins in all patients with approximately 70% of the patients administered beta blockers and ACE/ARB agents. Both studies were RCTs	2019 report: 1-RM knee extension tests, vastus lateralis diameter, FMD, inflammatory markers, and fasting glucose and insulin sensitivity	2019 & 2020 report: Acute and chronic (2x/week for 8 weeks) assessment of BFR Ex. during knee extension and flexion using a pneumatic cuff (23 cm wide) compressing the medium portion of each thigh 15–20 mm Hg greater than resting brachial systolic blood pressure. Cuffs remained inflated throughout the 3 sets of 8, 10, and 12 reps at 30–40% 1-RM with 45-s rest periods between sets during which the cuffs remained inflated. Each leg was exercised separately as described above with a cadence of 1-s for the concentric phase and 2-s for the eccentric phase. Aerobic Ex. at 60–80% of HRmax for 35 min 3x/week was also performed in the BFR group and was also performed in the control group	2019 & 2020 report: No adverse events were reported
		2020 report: HR, BP, NT-proBNP, Fibrinogen, and D-dimer		2019 report: BFR Ex. significantly increased muscle strength in the 1-RM and decreased systolic blood pressure with near significant improvements in FMD and insulin sensitivity
				2020 report: Acutely, BFR Ex. produced significantly greater HR, SBP, and DBP during each of the 3 sets compared to baseline measures, but both the SBP and DBP were lower after the third set compared to the second set. Post-exercise HR, SBP, and DBP were significantly lower than the measures after each of the 3 sets. Chronically, SBP was significantly lower post-BFR compared to the control group performing aerobic Ex. No significant changes were observed in NT-proBNP, Fibrinogen, and D-dimer values
Ishizaka et al. (2019)	6 males with surgically repaired valvular heart disease and 1 female with MR, AR, and heart failure with baseline EF range of 20–66% who participated in the study 105–1,018 days from diagnosis of valvular heart disease. Medications included ACE-I/ARB, Beta-blockers, and calcium channel blockers. The study design was a pre-post study without control group	Maximal voluntary isometric knee extension bilaterally, EMG amplitude of the rectus femoris, vastus lateralis, and vastus medialis muscles during both concentric and eccentric contractions performed bilaterally which were also summed and averaged. Subjective Borg RPE with and without BFR was also measured	Acute examination of EMG activity at 10 and 20% of 1-RM with and without BFR. BFR was administered using the KAATSU system with 60 mm wide cuffs placed proximally around both thighs while participants were seated on the knee extension machine. The cuff pressure was set at 180 mmHg and the cuffs remained inflated throughout rest periods and were deflated between each of the 4 test conditions. The 4 test conditions that were examined included 10 and 20% of 1-RM with and without BFR with patients performing 3 sets of 30 bilateral knee extensions with 30 s of rest between sets and 5 min of rest between conditions. After completing the first test condition of 10% 1-RM without BFR the remaining 3 test conditions were administered using a block-randomization procedure	No adverse events were reported. All males completed the protocol, but the woman was only able to complete the 10% 1-RM protocol. BFR at 10% 1-RM significantly increased EMG amplitude of all muscles in both concentric and eccentric phases which was not significantly greater at 20% of 1-RM. The RPE increased significantly with BFR at both intensities and the RPE at 20% 1-RM with and without BFR was significantly greater than at 10% of 1-RM. Age was significantly correlated to EMG amplitude at several concentric and eccentric phases without BFR, but no significant correlations were found with BFR.
Ogawa et al. (2021)	21 mostly male patients early after cardiac surgery for mostly valvular heart disease with NYHA class 2–3 were randomly assigned to a BFR group or control group. Both groups attended outpatient cardiac rehabilitation 2x/week for 12 weeks with the addition of BFR Ex. to the BFR group. The mean EF and BNP of the BFR and control group was 54 and 59% and 303 and 172, respectively. Four patients in each group had atrial fibrillation and approximately 70% of the patients in each group were hypertensive. Medications administered to patients were not listed, but it appears that patients received thrombolytic agents during the early phase of rehabilitation. The study design was a RCT.	Body weight and composition, blood biochemistry, maximal voluntary isometric contraction of the knee extensors and handgrip, muscle size, and adverse effects	Early and chronic examination of BFR and cardiac rehabilitation versus cardiac rehabilitation alone. BFR was administered 2x/week using the KAATSU system with cuffs placed proximally around both thighs and cuff pressure increased from 100 mmHg to 160–200 mmHg over a 2–3-week period. BFR Ex. started 5–7 days after surgery if patients were able to walk 200 m and consisted of bilateral knee extension and flexion and leg press. BFR Ex. was started at a low-intensity (a single set of 20 repetitions with 5–10 kg and 20–30 kg for knee extension and flexion and leg press, respectively) and was progressed to 3 sets of 30 repetitions for each exercise with a 30 s rest between sets at 20–30% of 1-RM. The Borg RPE was used to monitor and control exercise and was consistently kept below 15	No adverse events were reported and CPK and D-dimer were normal after the 12 weeks study period. Early after cardiac surgery the BFR group had significantly greater body weight, anterior mid-thigh muscle thickness, and skeletal muscle mass while the control group had no significant improvement. Compared to early after surgery upon completion of the 12-weeks study, the BFR group was found to have a significant increase in body weight, anterior mid-thigh muscle thickness, skeletal muscle mass, walking speed, and knee extensor strength while no significant change from early after cardiac surgery to completion of the study was found in the control group. Low functioning patients tended to increase functional performance more than high functioning patients

The results of the above studies are shown in Table 4. The Nakajima et al. study found a significantly greater cross-sectional area of the quadriceps, hamstring, and adductor muscles with significant increases in leg press and knee extension and flexion as well as increases in peak watts, watts at the anaerobic threshold, peak oxygen consumption, and oxygen consumption at the anaerobic threshold without change in blood pressure. (Nakajima et al., 2010). The study by Madarame observed a significantly greater heart rate and noradrenaline response compared to non-BFRE as well as a significantly greater D-dimer and CRP after BFRE compared to non-BFRE which were no longer statistically significant after plasma volume correction (suggesting that hemoconcentration was responsible for the significant increases in these measures). Plasma fibrinogen/fibrin degradation products were unchanged after both forms of exercise. (Madarame et al., 2013). The study by Fukuda et al. found that BFRE produced significantly greater EMG and Borg RPE during all sets compared to non-BFRE. (Fukuda et al., 2013). The study by Ishizaka et al. observed that BFRE at 10% 1-RM significantly increased EMG amplitude of all muscles in both concentric and eccentric phases which was not significantly greater at 20% of 1-RM. (Ishizaka et al., 2019). The RPE increased significantly with BFR at both intensities and the RPE at 20% 1-RM with and without BFRE was significantly greater than at 10% of 1-RM. Also, age was significantly correlated to EMG amplitude at several concentric and eccentric phases without BFRE, but no significant correlations were found with BFRE. The study by Ogawa et al. found CPK and D-dimer were normal after the 12-weeks study period. Early after cardiac surgery, the BFR group had significantly greater body weight, anterior mid-thigh muscle thickness, and skeletal muscle mass while the control group had no significant improvement. Compared to early after surgery upon completion of the 12-weeks study, the BFR group was found to have a significant increase in body weight, anterior mid-thigh muscle thickness, skeletal muscle mass, walking speed, and knee extensor strength while no significant change from early after cardiac surgery to completion of the study was found in the control group. Low functioning patients tended to increase functional performance more than high functioning patients. (Ogawa et al., 2021) (Table 4).

The two other studies of BFRE in HD were performed in Slovenia using the same patient population while using a pneumatic cuff that was placed at the medium portion of each thigh (Table 4). The acute and chronic effects of BFRE were examined in patients with ischemic HD who were randomized to BFR or control group. The BFRE group performed knee extension and flexion 2x/week for 8 weeks with 3 sets of 8, 10, and 12 repetitions at 30–40% of 1-RM with 45-s rest periods between sets during which the cuffs remained inflated. Each leg was exercised separately with a cadence of 1-s for the concentric phase and 2-s for the eccentric phase. The cuff pressure was inflated 15–20 mmHg above resting brachial systolic pressure. The control group and the BFRE group performed aerobic exercise at 60–80% of maximal heart rate for 35 min, 3x/week during the 8-weeks study period. In the chronic study, BFRE significantly increased muscle strength in the 1-RM and decreased systolic blood pressure with near significant improvements in flow mediated dilation and insulin sensitivity. In the acute study, BFRE produced significantly greater HR, SBP, and DBP during each of the 3 sets compared to baseline measures, but both the SBP and DBP were lower after the third set compared to the second set. Also, post-exercise HR, SBP, and DBP were significantly lower than the measures after each of the 3 sets and no significant changes were observed in NT-proBNP, Fibrinogen, and D-dimer values. Furthermore, in the chronic study, SBP was significantly lower post-BFR compared to the control group performing aerobic exercise alone (Table 4).

## Discussion

It is important to note that BFRE was performed safely in all of the above studies without report of an adverse event.^41-49^ This is an important finding given that a variety of patients with HD (CABG and valvular surgery, PTCA, non-ST segment elevation MI and ST-segment elevation MI, dilated cardiomyopathy, and heart failure) performed BFRE making the results of this systematic review more generalizable. However, the relatively small number of total subjects in this systematic review (*n* = 140; 74 with HD and 66 with HF) and the carefully selected subjects with the HF subjects having mild to moderate HF based on BNP values highlights the need for further investigation of BFRE in HD and HF.

The results of the chronic BFRE by Nakajima et al. appear to be most promising for patients with HD in view of the significantly greater circumferential surface area in the quadriceps, hamstring, and adductor muscles, significant increases in leg strength, as well as submaximal and maximal exercise and cardiorespiratory capacity after 3 months of twice weekly BFR exercise performed at 20–30% 1 RM with an initial set of 30 repetitions followed by 3 sets of 15 repetitions and a 60 s rest between sets. (Nakajima et al., 2010). Thus, a short exercise duration (30–60 s x 4 = 120–240 s) performed with a low frequency (2x/week) and workload (20–30% 1 RM) yielded important results commonly found after much longer and more frequent exercise performed at a higher intensity. (Nakajima et al., 2010). Similar results were observed in the other four studies of chronic BFRE in HD. (Madarame et al., 2013; Kambič et al., 2019; Kambič et al., 2021; Ogawa et al., 2021). These are important factors for patients with HD who may be unable to exercise at the intensity, duration, and frequency needed to elicit similar changes using aerobic or more traditional resistance training. In fact, the improvement from BFRE in strength, exercise, and cardiorespiratory capacity was very similar to that observed after aerobic exercise performed at a greater intensity, duration, and frequency. (Passino et al., 2006).

The results of the acute and chronic BFRE studies in HD are also important since plasma fibrinogen and fibrin degradation products were unchanged and after plasma volume correction, the D-dimer and CRP values were no longer statistically different than before BFR exercise highlighting the potential safety of BFRE in patients with HD. (Madarame et al., 2013; Kambič, 2020). Furthermore, 38% of the patient population in the Ogawa et al. study had atrial fibrillation which has been a potential concern when considering BFRE in patients with HD and HF. It is also important to note that all of the BFRE studies presented in Table 3, Table 4 were performed upright and not in a supine position with all but one study performing BFRE in the lower extremities bilaterally with the one study not performing BFRE to the lower extremities performing BFRE in the upper extremities bilaterally. The use of bilateral upper extremity BFRE in patients with HD is likely to elicit a greater cardiovascular response than found in lower extremity BFRE and requires further investigation in patients with HD. One possible alternative may be unilateral versus bilateral BFRE in patients with HD or HF in need of improving upper extremity strength and endurance.

Although no studies of BFRE in HD utilized aerobic exercise training, the results of BFRE in HF are promising especially since aerobic BFRE appears to elicit a less aggressive hemodynamic response. (May et al., 2017; Tanaka and Takarada, 2018). Furthermore, the results of bilateral lower extremity BFRE with a cyclical occlusion and reperfusion protocol may elicit similar findings to those we observed in patients with HF. (Gempel et al., 2022; Johnson et al., 2022). Such effects could improve cardiac rehabilitation efforts by prescribing lower intensity exercise and eliciting a favorable cardiovascular response while increasing skeletal muscle strength and hypertrophy and possibly improve cardiac performance. However, further investigation of the effects of cyclical occlusion and reperfusion on skeletal muscle strength and hypertrophy as well as cardiovascular function in patients with HD and HF is needed. Despite this, the methods employed in the studies included in this systematic review that resulted in safe BFRE with significant improvements in many MHCHF components have been listed in Table 5 along with other factors that are considered important in the rehabilitation of patients with HD and HF. (Ambrosetti et al., 2020; Hansen et al., 2022). Although the suggested methods require further investigation and testing, they provide a framework to apply BFRE in the rehabilitation of patients with HD and HF. Limitations to this systematic review include the relatively small number of total subjects and the carefully selected subjects with the HF subjects having mild to moderate HF based on BNP values highlighting the need for further investigation of BFRE in HD and HF. Furthermore, investigation of feasibility, acceptability, adherence, adverse effects, and symptoms during and after BFRE is needed since very few studies have examined these important issues comprehensively in patients with HD and HF.

**TABLE 5:** Suggested methods to perform blood flow restriction training safely in patients with heart disease and heart failure.

1. Review risk factors for potential reasons to not perform BFRE including unstable or uncontrolled heart disease or heart failure, rapid and uncontrolled cardiac dysrhythmias, severe pulmonary hypertension or severe cardiac disease (valvular heart disease, myocarditis, endocarditis, pericarditis), history of venous thromboembolism, severe varicose veins, uncontrolled hypertension (> 180/110 mmHg), and an acute systemic illness
2. Discuss with the patient functional limitations and activities of daily living that are difficult to perform to target muscle groups in need of strengthening and aerobic conditioning
3. Obtain the resting heart rate, electrocardiogram (ECG), blood pressure, respiratory rate, rating of perceived exertion (RPE), symptoms, appearance and possibly girth measurements of the targeted extremities in sitting and/or supine. Examine the targeted extremities for signs, symptoms, and history of venous stasis and venous thrombosis
4. Educate patients about the procedures involved with blood flow restriction exercise
5. Determine the 1 repetition maximum (1-RM) using one of several different methods for the targeted muscle groups and repeat 1-RM measurements weekly or every 2–4 weeks to progress BFR resistance exercise. Determine peak oxygen consumption to prescribe aerobic BFRE at a specific percentage of the peak level and possibly use a percentage of age-predicted maximal heart rate and heart rate reserve if measurement of peak oxygen consumption is not possible
6. Apply the blood flow restriction cuff to one or both of the targeted proximal extremities and inflate it to the desired limb occlusion pressure
7. Obtain the post-cuff inflation heart rate, blood pressure, respiratory rate, rating of perceived exertion, symptoms, ECG, and appearance of the targeted extremity or extremities and compare to the values obtained in sitting and/or supine
8. Perform exercise with the inflated blood flow restriction cuff to the targeted extremity while continuously monitoring symptoms and the ECG, and measuring the heart rate, blood pressure, respiratory rate, rating of perceived exertion, and appearance of the exercising extremity after each set of exercise and compare to resting values and each set of exercise. a. Blood flow restriction resistance training: 3–4 sets of 15–30 repetitions at 20–30% of 1-RM with 30–60 s rest periods between sets, 2–3x/week
b. Blood flow restriction aerobic training: Aerobic exercise such as treadmill ambulation or cycle ergometry performed at 40–70% of peak oxygen consumption for 10–15 min, 2–3x/week
9. Deflate and remove the blood flow restriction cuff and obtain the symptoms, heart rate, blood pressure, respiratory rate, rating of perceived exertion, ECG, appearance and possibly girth measurements of the targeted extremity and compare to the values obtained in sitting and/or supine
10. Terminate BFRE if any of the following occur including: a) symptoms associated with heart disease (angina, dyspnea, dizziness, etc.) or heart failure (dyspnea and fatigue), b) a hypertensive or hypotensive blood pressure response, c) ECG abnormalities, d) an abnormal heart rate, respiratory rate, or RPE response, d) marked peripheral edema in targeted extremity, e) signs or symptoms of venous stasis or venous thrombosis

In summary, BFRE in HD and HF was performed safely and was observed to improve one or more of the following measures in the reviewed studies including skeletal muscle strength, endurance, and hypertrophy; cardiorespiratory performance; mitochondrial function; exercise tolerance; functional performance; immune humoral function; and possibly cardiac performance. (Nakajima et al., 2010; Fukuda et al., 2013; Madarame et al., 2013; Tanaka and Takarada, 2018; Groennebaek et al., 2019; Ishizaka et al., 2019; Kambič et al., 2019; Kambič et al., 2021; Ogawa et al., 2021). Although these results are promising, further investigation of BFRE in patients with HD and HF is needed in view of the beneficial effects on skeletal muscle, functional performance, and cardiorespiratory function in older subjects without heart disease. (Hughes et al., 2017; Beckwée et al., 2019; Centner et al., 2019; Van Cant et al., 2020; Nitzsche et al., 2021; Rodrigo-Mallorca et al., 2021; Labata-Lezaun et al., 2022). Finally, there is a distinct need for additional randomized controlled trials examining the effects of BFRE in patients with HD and HF.

## Conclusion

In view of the above systematic review, BFRE has been performed safely with no report of adverse event in patients with a variety of different types of HD and in patients with HF. The available literature suggests that many components of the MHCHF are improved with BFRE which may attenuate its viscous cycle. The components of the MHCHF that can be potentially improved with BFRE include left ventricular dysfunction, inflammatory markers, inactivity, a catabolic state, skeletal and possibly respiratory muscle myopathy, dyspnea and fatigue, ANS activity, and peripheral blood flow. Although the currently available BFRE literature has demonstrated improvements in each of these components in patients with HD and HF, further investigation of the role BFRE may have in the management of HD and HF is needed.

## Data Availability

The original contributions presented in the study are included in the article/Supplementary Material, further inquiries can be directed to the corresponding author.

## References

[B1] Ambrosetti M. Abreu A. Corrà U. Davos C. H. Hansen D. Frederix I. (2020). Secondary Prevention through Comprehensive Cardiovascular Rehabilitation: From Knowledge to Implementation. 2020 Update. A Position Paper from the Secondary Prevention and Rehabilitation Section of the European Association of Preventive Cardiology. Eur. J. Prev. Cardiol. 28, 460–495. 10.1177/2047487320913379 33611446

[B2] BeckwéeD. DelaereA. DelaereA. AelbrechtS. BaertV. BeaudartC. (2019). Exercise Interventions for the Prevention and Treatment of Sarcopenia. A Systematic Umbrella Review. J. Nutr. Health Aging 23 (6), 494–502. 10.1007/s12603-019-1196-8 31233069

[B3] Bordessa J. M. Hearn M. C. Reinfeldt A. E. Smith T. A. Baweja H. S. Levy S. S. (2021). Comparison of Blood Flow Restriction Devices and Their Effect on Quadriceps Muscle Activation. Phys. Ther. Sport 49, 90–97. 10.1016/j.ptsp.2021.02.005 33647529

[B4] Centner C. Wiegel P. Gollhofer A. König D. (2019). Effects of Blood Flow Restriction Training on Muscular Strength and Hypertrophy in Older Individuals: A Systematic Review and Meta-Analysis. Sports Med. 49 (1), 95–108. 10.1007/s40279-018-0994-1 30306467 PMC6349784

[B5] Chen C.-Y. Bonham A. C. (2010). Postexercise Hypotension. Exerc Sport Sci. Rev. 38 (3), 122–127. 10.1097/JES.0b013e3181e372b5 20577060 PMC2936915

[B6] Christiansen D. Eibye K. H. Hostrup M. Bangsbo J. (2019). Blood Flow-Restricted Training Enhances Thigh Glucose Uptake during Exercise and Muscle Antioxidant Function in Humans. Metabolism 98, 1–15. 10.1016/j.metabol.2019.06.003 31199953

[B7] Christiansen D. Eibye K. Hostrup M. Bangsbo J. (2020). Training with Blood Flow Restriction Increases Femoral Artery Diameter and Thigh Oxygen Delivery during Knee‐Extensor Exercise in Recreationally Trained Men. J. Physiol. 598 (12), 2337–2353. 10.1113/jp279554 32246768

[B8] Coats A. J. S. Clark A. L. Piepoli M. Volterrani M. Poole-Wilson P. A. (1994). Symptoms and Quality of Life in Heart Failure: The Muscle Hypothesis. Heart 72 (2 Suppl. l), S36–S39. 10.1136/hrt.72.2_suppl.s36 PMC1025572 7946756

[B9] Coats A. (1996). The "Muscle Hypothesis" of Chronic Heart Failure. J. Mol. Cell. Cardiol. 28 (11), 2255–2262. 10.1006/jmcc.1996.0218 8938579

[B10] Crisafulli A. de Farias R. R. Farinatti P. Lopes K. G. Milia R. Sainas G. (2018). Blood Flow Restriction Training Reduces Blood Pressure during Exercise without Affecting Metaboreflex Activity. Front. Physiol. 9, 1736. 10.3389/fphys.2018.01736 30618781 PMC6299290

[B11] Fukuda T. Yasuda T. Fukumura K. Iida H. Morita T. Sato Y. (2013). Low-Intensity Kaatsu Resistance Exercises Using an Elastic Band Enhance Muscle Activation in Patients with Cardiovascular Diseases. Int. J. KAATSU Ttaining Res. 9, 1–5. 10.3806/ijktr.9.1

[B12] Gempel S. M. Johnson A. N. Swick R. M. Hecht M. J. Feeback S. N. Garcia T. (2022). CSM 2022 Cardiovascular and Pulmonary Abstracts. Cardiopulm. Phys. Ther. J. 33 (1), e12–e31. 10.1097/cpt.0000000000000198

[B13] Groennebaek T. Sieljacks P. Nielsen R. Pryds K. Jespersen N. R. Wang J. (2019). Effect of Blood Flow Restricted Resistance Exercise and Remote Ischemic Conditioning on Functional Capacity and Myocellular Adaptations in Patients with Heart Failure. Circ. Heart Fail. 12 (12), e006427. 10.1161/circheartfailure.119.006427 31830830

[B14] Halliwill J. R. (2001). Mechanisms and Clinical Implications of Post-Exercise Hypotension in Humans. Exerc. Sport Sci. Rev. 29 (2), 65–70. 10.1097/00003677-200104000-00005 11337825

[B15] Hansen D. Abreu A. Ambrosetti M. Cornelissen V. Gevaert A. Kemps H. (2022). Exercise Intensity Assessment and Prescription in Cardiovascular Rehabilitation and Beyond: Why and How: A Position Statement from the Secondary Prevention and Rehabilitation Section of the European Association of Preventive Cardiology. Eur. J. Prev. Cardiol. 29 (1), 230–245. 10.1093/eurjpc/zwab007 34077542

[B16] Higashi Y. Yoshizumi M. (2004). Exercise and Endothelial Function: Role of Endothelium-Derived Nitric Oxide and Oxidative Stress in Healthy Subjects and Hypertensive Patients. Pharmacol. Ther. 102 (1), 87–96. 10.1016/j.pharmthera.2004.02.003 15056500

[B17] Horiuchi M. Okita K. (2012). Blood Flow Restricted Exercise and Vascular Function. Int. J. Vasc. Med. 2012, 543218. 10.1155/2012/543218 23133756 PMC3485988

[B18] Hughes L. Paton B. Rosenblatt B. Gissane C. Patterson S. D. (2017). Blood Flow Restriction Training in Clinical Musculoskeletal Rehabilitation: A Systematic Review and Meta-Analysis. Br. J. Sports Med. 51 (13), 1003–1011. 10.1136/bjsports-2016-097071 28259850

[B19] Ishizaka H. Uematsu A. Mizushima Y. Nozawa N. Katayanagi S. Matsumoto K. (2019). Blood Flow Restriction Increases the Neural Activation of the Knee Extensors during Very Low-Intensity Leg Extension Exercise in Cardiovascular Patients: A Pilot Study. J. Clin. Med. 8 (8), 1252. 10.3390/jcm8081252 PMC6723568 31430903

[B20] Johnson A. N. Swick R. M. Hecht M. J. Feeback S. N. Gempel S. M. Garcia T. (2022). CSM 2022 Cardiovascular and Pulmonary Abstracts. Cardiopulm. Phys. Ther. J. 33 (1), e12–e31. 10.1097/cpt.0000000000000198

[B21] Kambič T. (2020). Blood Flow Restriction Training: You Can Occlude Your Veins, but Not Your Oxygen Transport. J. Physiol. 598 (18), 3825–3826. 10.1113/jp279936 32539161

[B22] Kambic T. Jug B. Piepoli M. F. Lainscak M. (2022). Is Blood Flow Restriction Resistance Training the Missing Piece in Cardiac Rehabilitation of Frail Patients? Eur. J. Prev. Cardiol., zwac048. 10.1093/eurjpc/zwac048 35253869

[B23] Kambič T. Novaković M. Tomažin K. Strojnik V. Božič-Mijovski M. Jug B. (2021). Hemodynamic and Hemostatic Response to Blood Flow Restriction Resistance Exercise in Coronary Artery Disease. J. Cardiovasc Nurs. 36 (5), 507–516. 10.1097/jcn.0000000000000699 32496365

[B24] Kambič T. Novaković M. Tomažin K. Strojnik V. Jug B. (2019). Blood Flow Restriction Resistance Exercise Improves Muscle Strength and Hemodynamics, but Not Vascular Function in Coronary Artery Disease Patients: A Pilot Randomized Controlled Trial. Front. Physiol. 10, 656. 10.3389/fphys.2019.00656 31244668 PMC6581774

[B25] Labata-Lezaun N. Llurda-Almuzara L. González-Rueda V. López-de-Celis C. Cedeño-Bermúdez S. Bañuelos-Pago J. (2022). Effectiveness of Blood Flow Restriction Training on Muscle Strength and Physical Performance in Older Adults: A Systematic Review and Meta-Analysis. Archives Phys. Med. Rehabilitation S0003-9993 (22), 00004–1. 10.1016/j.apmr.2021.12.015 35026149

[B26] Li S. Li S. Wang L. Quan H. Yu W. Li T. (2022). The Effect of Blood Flow Restriction Exercise on Angiogenesis-Related Factors in Skeletal Muscle Among Healthy Adults: A Systematic Review and Meta-Analysis. Front. Physiol. 13, 814965. 10.3389/fphys.2022.814965 35250618 PMC8892188

[B27] Liu Y. Jiang N. Pang F. Chen T. (2021). Resistance Training with Blood Flow Restriction on Vascular Function: A Meta-Analysis. Int. J. Sports Med. 42 (7), 577–587. 10.1055/a-1386-4846 33735919

[B28] Loenneke J. P. Fahs C. A. Rossow L. M. Abe T. Bemben M. G. (2012). The Anabolic Benefits of Venous Blood Flow Restriction Training May Be Induced by Muscle Cell Swelling. Med. Hypotheses 78 (1), 151–154. 10.1016/j.mehy.2011.10.014 22051111

[B29] Madarame H. Kurano M. Fukumura K. Fukuda T. Nakajima T. (2013). Haemostatic and Inflammatory Responses to Blood Flow-Restricted Exercise in Patients with Ischaemic Heart Disease: A Pilot Study. Clin. Physiol. Funct. Imaging 33 (1), 11–17. 10.1111/j.1475-097X.2012.01158.x 23216760

[B30] Masri B. A. Eisen A. Duncan C. P. McEwen J. A. (2020). Tourniquet-induced Nerve Compression Injuries Are Caused by High Pressure Levels and Gradients - a Review of the Evidence to Guide Safe Surgical, Pre-hospital and Blood Flow Restriction Usage. BMC Biomed. Eng. 2, 7. 10.1186/s42490-020-00041-5 32903342 PMC7422508

[B31] May A. K. Brandner C. R. Warmington S. A. (2017). Hemodynamic Responses Are Reduced with Aerobic Compared with Resistance Blood Flow Restriction Exercise. Physiol. Rep. 5 (3), e13142. 10.14814/phy2.13142 28183863 PMC5309582

[B32] May A. K. Russell A. P. Della Gatta P. A. Warmington S. A. (2022). Muscle Adaptations to Heavy-Load and Blood Flow Restriction Resistance Training Methods. Front. Physiol. 13, 837697. 10.3389/fphys.2022.837697 35185627 PMC8850930

[B33] Mostoufi B. DaSilva C. Bell E. Smith M. Gempel S. Bell N. (2020). Optimal Limb Occlusion Pressure for Blood Flow Restriction in Heart Failure and Health: Case Comparison. J. Acute Care Phys. Ther. https://apta.confex.com/apta/csm2020/meetingapp.cgi/Paper/25695

[B34] Murray J. Bennett H. Boyle T. Williams M. Davison K. (2021). Approaches to Determining Occlusion Pressure for Blood Flow Restricted Exercise Training: Systematic Review. J. Sports Sci. 39 (6), 663–672. 10.1080/02640414.2020.1840734 33135570

[B35] Nakajima T. Kurano M. Sakagami F. Iida H. Fukumura K. Fukuda T. (2010). Effects of Low-Intensity KAATSU Resistance Training on Skeletal Muscle Size/strength and Endurance Capacity in Patients with Ischemic Heart Disease. Int. J. KAATSU Ttaining Res. 6 (1), 1–7. 10.3806/ijktr.6.1

[B36] NIH (2021). Quality Assessment Tool for Before-After (Pre-Post) Studies with No Control Group. National Heart, Lung and Blood Institute [Online]. Available: https://www.nhlbi.nih.gov/health-topics/study-quality-assessment-tools (Accessed May 20, 2022).

[B37] Nitzsche N. Stäuber A. Tiede S. Schulz H. (2021). The Effectiveness of Blood-Flow Restricted Resistance Training in the Musculoskeletal Rehabilitation of Patients with Lower Limb Disorders: A Systematic Review and Meta-Analysis. Clin. Rehabil. 35 (9), 1221–1234. 10.1177/02692155211003480 33749352

[B38] Ogawa H. Nakajima T. Shibasaki I. Nasuno T. Kaneda H. Katayanagi S. (2021). Low-Intensity Resistance Training with Moderate Blood Flow Restriction Appears Safe and Increases Skeletal Muscle Strength and Size in Cardiovascular Surgery Patients: A Pilot Study. Jcm 10 (3), 547. 10.3390/jcm10030547 33540756 PMC7867301

[B39] Passino C. Severino S. Poletti R. Piepoli M. F. Mammini C. Clerico A. (2006). Aerobic Training Decreases B-type Natriuretic Peptide Expression and Adrenergic Activation in Patients with Heart Failure. J. Am. Coll. Cardiol. 47 (9), 1835–1839. 10.1016/j.jacc.2005.12.050 16682309

[B40] Patterson S. D. Ferguson R. A. (2011). Enhancing Strength and Postocclusive Calf Blood Flow in Older People with Training with Blood-Flow Restriction. J. Aging Phys. Act. 19 (3), 201–213. 10.1123/japa.19.3.201 21727301

[B41] Piepoli M. F. Coats A. J. S. (2013). The 'skeletal Muscle Hypothesis in Heart Failure' Revised. Eur. Heart J. 34 (7), 486–488. 10.1093/eurheartj/ehs463 23297313

[B42] Pignanelli C. Christiansen D. Burr J. F. (2021). Blood Flow Restriction Training and the High-Performance Athlete: Science to Application. J. Appl. Physiology (1985) 130 (4), 1163–1170. 10.1152/japplphysiol.00982.2020 33600282

[B43] Pinto R. R. Karabulut M. Poton R. Polito M. D. (2018). Acute Resistance Exercise with Blood Flow Restriction in Elderly Hypertensive Women: Haemodynamic, Rating of Perceived Exertion and Blood Lactate. Clin. Physiol. Funct. Imaging 38 (1), 17–24. 10.1111/cpf.12376 27283375

[B44] Pinto R. R. Polito M. D. (2016). Haemodynamic Responses during Resistance Exercise with Blood Flow Restriction in Hypertensive Subjects. Clin. Physiol. Funct. Imaging 36 (5), 407–413. 10.1111/cpf.12245 26095652

[B45] Reina-Ruiz Á. J. Galán-Mercant A. Molina-Torres G. Merchán-Baeza J. A. Romero-Galisteo R. P. González-Sánchez M. (2022). Effect of Blood Flow Restriction on Functional, Physiological and Structural Variables of Muscle in Patients with Chronic Pathologies: A Systematic Review. Ijerph 19 (3), 1160. 10.3390/ijerph19031160 35162182 PMC8835162

[B46] Rodrigo-Mallorca D. Loaiza-Betancur A. F. Monteagudo P. Blasco-Lafarga C. Chulvi-Medrano I. (2021). Resistance Training with Blood Flow Restriction Compared to Traditional Resistance Training on Strength and Muscle Mass in Non-Active Older Adults: A Systematic Review and Meta-Analysis. Ijerph 18 (21), 11441. 10.3390/ijerph182111441 34769957 PMC8583588

[B47] Rossow L. M. Fahs C. A. Sherk V. D. Seo D.-I. Bemben D. A. Bemben M. G. (2011). The Effect of Acute Blood-Flow-Restricted Resistance Exercise on Postexercise Blood Pressure. Clin. Physiol. Funct. Imaging 31 (6), 429–434. 10.1111/j.1475-097X.2011.01038.x 21981453

[B48] Shimizu R. Hotta K. Yamamoto S. Matsumoto T. Kamiya K. Kato M. (2016). Low-intensity Resistance Training with Blood Flow Restriction Improves Vascular Endothelial Function and Peripheral Blood Circulation in Healthy Elderly People. Eur. J. Appl. Physiol. 116 (4), 749–757. 10.1007/s00421-016-3328-8 26822582

[B49] Shweiki D. Itin A. Soffer D. Keshet E. (1992). Vascular Endothelial Growth Factor Induced by Hypoxia May Mediate Hypoxia-Initiated Angiogenesis. Nature 359 (6398), 843–845. 10.1038/359843a0 1279431

[B50] Silva J. C. G. Pereira Neto E. A. Pfeiffer P. A. S. Neto G. R. Rodrigues A. S. Bemben M. G. (2019). Acute and Chronic Responses of Aerobic Exercise with Blood Flow Restriction: A Systematic Review. Front. Physiol. 10, 1239. 10.3389/fphys.2019.01239 31636569 PMC6787286

[B51] Smart N. A. Waldron M. Ismail H. Giallauria F. Vigorito C. Cornelissen V. (2015). Validation of a New Tool for the Assessment of Study Quality and Reporting in Exercise Training Studies. Int. J. Evid. Based Healthc. 13 (1), 9–18. 10.1097/xeb.0000000000000020 25734864

[B52] Takano H. Morita T. Iida H. Asada K.-I. Kato M. Uno K. (2005). Hemodynamic and Hormonal Responses to a Short-Term Low-Intensity Resistance Exercise with the Reduction of Muscle Blood Flow. Eur. J. Appl. Physiol. 95 (1), 65–73. 10.1007/s00421-005-1389-1 15959798

[B53] Tanaka Y. Takarada Y. (2018). The Impact of Aerobic Exercise Training with Vascular Occlusion in Patients with Chronic Heart Failure. Esc. Heart Fail. 5 (4), 586–591. 10.1002/ehf2.12285 29575708 PMC6073027

[B54] Van Cant J. Dawe-Coz A. Aoun E. Esculier J.-F. (2020). Quadriceps Strengthening with Blood Flow Restriction for the Rehabilitation of Patients with Knee Conditions: A Systematic Review with Meta-Analysis. Bmr 33 (4), 529–544. 10.3233/bmr-191684 32310159

[B55] Weatherholt A. M. Vanwye W. R. Lohmann J. Owens J. G. (2019). The Effect of Cuff Width for Determining Limb Occlusion Pressure: A Comparison of Blood Flow Restriction Devices. Int. J. Exerc Sci. 12 (3), 136–143. 30761200 10.70252/RWVU7100 PMC6355123

[B56] Wong M. L. Formiga M. F. Owens J. Asken T. Cahalin L. P. (2018). Safety of Blood Flow Restricted Exercise in Hypertension: A Meta-Analysis and Systematic Review with Potential Applications in Orthopedic Care 33 (2), 80–88. 10.1097/bto.0000000000000288

[B57] Wong V. Song J. S. Bell Z. W. Yamada Y. Spitz R. W. Abe T. (2021). Blood Flow Restriction Training on Resting Blood Pressure and Heart Rate: A Meta-Analysis of the Available Literature. J. Hum. Hypertens.. 10.1038/s41371-021-00561-0 34140637

