# Supplementary material for: Beneficial Role of Blood Flow Restriction Exercise in Heart Disease and Heart Failure Using the Muscle Hypothesis of Chronic Heart Failure and a Growing Literature
Source: Front Physiol. 2022 Jul 6;13:924557. doi: 10.3389/fphys.2022.924557 (PMC9296815; doi:10.3389/fphys.2022.924557)
Supplement: Supplementary file 1 [file Table1.docx]

**APPENDIX 1**

The following clinical question and its respective concept map were used to develop the search strategy: How safe is blood flow restriction training (BFR) and what is its effectiveness on cardiorespiratory fitness, functional performance, systemic biomarkers, and skeletal muscle mass and strength in subjects with diagnosed heart disease and / or heart failure?

| P: Older adults with diagnosed heart disease and / or heart failure | I: BFR | C: - | O: Cardiorespiratory fitness, functional performance, systemic biomarkers, skeletal muscle mass and strength |
| --- | --- | --- | --- |
| Cardiovascular Disease (MeSH)  Heart Diseases (MeSH) | Resistance Training (MeSH)  Exercise Therapy (MeSH)  Aerobic Exercise (MeSH)  Blood Flow Restriction (Keyword)  Kaatsu Training (Keyword)  Vascular Occlusion (Keyword) |  | Oxygen consumption (MeSH)  Physical Fitness (MeSH)  Functional performance (Keyword)  Muscle, Skeletal (MeSH)  Muscle Mass (Keyword)  Muscle Strength (MeSH)  Biomarkers (MeSH) |
| Heart Failure (MeSH) |  |  |  |
|  |  |  |  |
|  |  |  |  |

PubMed search:

((((cardiovascular disease[MeSH Terms]) OR (heart diseases[MeSH Terms])) OR (heart failure[MeSH Terms])) AND ((((((resistance training[MeSH Terms]) OR (exercise therapy[MeSH Terms])) OR (aerobic exercise[MeSH Terms])) AND ("blood flow restriction")) OR ("kaatsu training")) OR ("vascular occlusion"))) AND (((((((oxygen consumption[MeSH Terms]) OR (physical fitness[MeSH Terms])) OR ("functional performance")) OR (muscle, skeletal[MeSH Terms])) OR ("muscle mass")) OR (muscle strength[MeSH Terms])) OR (biomarkers[MeSH Terms]))

Cochrane search:

#1 MeSH descriptor: [Cardiovascular Diseases]

#2 MeSH descriptor: [Heart Diseases]

#3 MeSH descriptor: [Heart Failure]

#4 MeSH descriptor: [Resistance Training]

#5 MeSH descriptor: [Exercise Therapy]

#6 MeSH descriptor: [Exercise]

#7 (Blood Flow Restriction):ti,ab,kw

#8 (Blood Flow Restricted):ti,ab,kw

#9 (Kaatsu):ti,ab,kw

#10 (Vascular Occlusion):ti,ab,kw

#11 MeSH descriptor: [Oxygen Consumption]

#12 MeSH descriptor: [Physical Fitness]

#13 (Functional performance):ti,ab,kw

#14 MeSH descriptor: [Muscle, Skeletal]

#15 (Muscle Mass):ti,ab,kw

#16 MeSH descriptor: [Muscle Strength]

#17 MeSH descriptor: [Biomarkers]

#18 #1 or #2 or #3

#19 #4 or #5 or #6

#20 #7 or #8 or #9 or #10

#21 #19 and #20

#22 #11 or #12 or #13 or #14 or #15 or #16 or #17

#23 #18 and #21 and #22
